# Acute Lymphoid Leukemia Cells with Greater Stem Cell Antigen-1 (Ly6a/Sca-1) Expression Exhibit Higher Levels of Metalloproteinase Activity and Are More Aggressive In Vivo

**DOI:** 10.1371/journal.pone.0088966

**Published:** 2014-02-20

**Authors:** Yu-Chiao Hsu, Kurt Mildenstein, Kordell Hunter, Olena Tkachenko, Craig A. Mullen

**Affiliations:** Department of Pediatrics, University of Rochester, Rochester, New York, United States of America; B.C. Cancer Agency, Canada

## Abstract

Stem cell antigen-1 (Ly6a/Sca-1) is a gene that is expressed in activated lymphocytes, hematopoietic stem cells and stem cells of a variety of tissues in mice. Despite decades of study its functions remain poorly defined. These studies explored the impact of expression of this stem cell associated gene in acute lymphoid leukemia. Higher levels of Ly6a/Sca-1 expression led to more aggressive leukemia growth in vivo and earlier death of hosts. Leukemias expressing higher levels of Ly6a/Sca-1 exhibited higher levels of matrix metalloproteinases. The results suggest the hypothesis that the more aggressive behavior of Ly6a/Sca-1 expressing leukemias is due at least in part to greater capacity to degrade microenvironmental stroma and invade tissues.

## Introduction

We recently discovered that acute lymphoid leukemia cells increase expression of some genes in vivo in the allogeneic environment, many of which are related to immune function [1]. One of these genes is lymphocyte antigen 6 locus A, Ly6a, originally discovered in the 1970s in activated lymphocytes [2], [3]. In a parallel literature, stem cell antigen-1 (Sca-1) was described as a cell surface marker on hematopoietic and other tissue stem cells [4], [5]. Work in the 1980’s demonstrated the molecular identity of Ly6a and Sca-1[6]–[8]. The exact functions of Ly6a/Sca-1 remain unknown. It is a glycosylphosphatidylinositol (GPI)-anchored protein present in a complex cell-surface lipid raft and likely functions as a coregulator of lipid raft mediated cell signaling [9], [10]. Although Ly6a/Sca-1 does bind some cells including B and T lymphocytes no ligand has been molecularly identified [11]–[13]. An excellent review was published in 2007 that highlights the molecule’s roles in immune function, hematopoiesis and stem cell biology [14].

The work reported here tested the hypothesis that increased expression of Ly6a/Sca-1 by lymphoid leukemia cells promotes increased aggressiveness in vivo. We compared the growth of high and low Ly6a/Sca-1 expressing leukemia cells in vivo. We discovered that higher levels of Ly6a/Sca-1 expression led to more aggressive growth in vivo and reduced survival for hosts. Moreover we observed that leukemias expressing higher levels of Ly6a/Sca-1 exhibited higher levels of matrix metalloproteinases.

## Materials and Methods

This study was carried out in strict accordance with the recommendations in the Guide for the Care and Use of Laboratory Animals of the National Institutes of Health. The protocol was approved by the Committee on the Ethics of Animal Experiments of the University of Rochester (UCAR approved protocol 100258/2003-237).

### Cell Lines

C1498 (ATCC) is a spontaneous C57BL/6 acute NKT cell leukemia [15]. NSTY1 is a C57BL/6 murine pre-B acute lymphoblastic leukemia (ALL) that has an INK/ARF region deletion and is driven by the human p210 bcr/abl oncogene [16]. Leukemia clones were derived by limiting dilution cultures. ASLN is a pre-B ALL C57BL/6 murine cell line driven by a human p190 bcr/abl oncogene [16]. Cells were propagated in RPMI with 10% fetal calf serum. In experiments assessing inducibility of Ly6a/Sca-1 recombinant murine interferon-gamma at 10 ng/ml was added to cultures.

### Retroviral and Lentiviral Vectors

Plasmids PM4 (which encodes a retroviral vector containing the cDNA for Ly6a/Sca-1 as well as the eGFP and zeocin resistance gene cDNAs) and pTJ66 (the control plasmid which encodes a retroviral vector that contains eGFP and zeocin resistance cDNAs) were gifts of Dr. G. K. Pavlath of Emory University [17]. Retrovirus was produced by lipofectamine facilitated transient transfection of helper-free retroviral vector producer Phoenix cell lines with plasmids PM4 or pTJ66. C1498 leukemia cells were exposed to retroviral vector supernatant with polybrene 5 µg/ml; following 700 g centrifugation at room temperature they were incubated overnight at 37°C, and then grown in zeocin 300 µg/ml to select for vector expressing cells. Firefly luciferase cDNA was transferred to leukemia cells using a lentiviral vector and transduced cells were selected by geneticin. To produce Ly6a/Sca-1 knockdown cells lines NSTY or ASLN leukemia cells were treated with Ly6a/Sca-1 specific shRNA lentivirus vectors (Sigma Aldrich) or control nontarget shRNA lentivirus per manufacturer protocol and then selected in puromycin 1 µg/ml.

### In Vivo Assays

Female C57BL/6 mice were intravenously injected in the tail vein with leukemia cells in 200 µl Hank’s balanced salt solution. The cell number in experiments is specified in figure legends. Mice were examined daily for signs of illness and euthanized when they appeared moribund. Femur bone marrow and/or spleen were harvested in some cases for flow cytometric assessment of Ly6a/Sca-1 expression. In some experiments assessing growth after allogeneic transplant C57BL/6 mice were given 800 cGy total body irradiation (given in two equal divided doses 14–16 hours apart) and intraperitoneal 5-fluoruracil (0.5 mg) and a day later were infused with 4×10^6^ marrow cells plus 10×10^6^ splenocytes from C3.SW mice. 1×10^6^ leukemia cells (C1498-Sca or C1498-vector only) were mixed with the graft cells immediately prior to cell infusion. C57BL/6 and C3.SW mice are MHC antigen matched (H2b) but minor histocompatibility antigen mismatched at many loci (H1, H3, H7, H8, H9, H13) [18], [19]. C3.SW are H2b and were derived from an 11 generation back cross of C3H against a non-inbred H2b donor strain. In some experiments doxycycline was added to the drinking water at a final concentration of 0.5 mg/ml; mice drank ad lib.

### In Vivo Bioluminescent Imaging

Detection of leukemia cells with luciferase signals were performed by the IVIS ImagingSystem (Xenogen, Alameda, CA). Images and measurements of bioluminescent signals were acquired and analyzed using Living Image software (Xenogen). The animals were injected with D-luciferin (Gold Biotechnology) at 150 mg/kg in DPBS by i.p. 10 minutes before acquiring the images. Bioluminescence under 5×10^4^ photons/sec was considered background.

### Flow Cytometry

Ly6a/Sca-1 expression was detected using the D7 monoclonal antibody (BD Pharmingen). Rat IgG2a isotype controls were used. Analysis was performed with either WinMDI 2.8 software or Winlist 6.0 software.

### Western Blot

1×10^6^ cells were lysed using RIPA buffer (Thermo Fisher). Protein concentrations were measured with the BCA protein reagent (Pierce Chemical). 20 µg of protein/lane samples were run on a 10% polyacrylamide gel with a Tris/glycine running buffer system and then transferred onto a PVDF membrane. The blots were probed with MMP9 (Abcam) and beta-actin (Santa Cruz Biotechnology) antibodies at 4°C overnight. The secondary antibody [rabbit or mouse anti-goat IgG (Santa Cruz Biotechnology) was used at room temperature for 1 hr. Immunoblot analyses were performed with horseradish peroxidase-conjugated anti-rabbit or anti-mouse IgG antibodies using enhanced chemiluminescence Western blotting detection reagents (Amersham Biosciences). Beta-actin was used as a control.

### Matrix Metalloproteinase (MMP) Assay

Leukemia cell lines were lysed with RIPA buffer (Sigma) following the manufacturer’s instructions. Protein concentrations of the lysates were measured with the BCA protein reagent (Pierce Chemical). 20 µl of lysate was added to 80 µl MMP buffer (10 mM Tris pH 7.5, 150 mM NaCl, 10 mMCaCl2, 0.05% Triton X-100). Fluorogenic peptide substrate IX (R&D Systems) was added to a final concentration of 10 µM. Samples were read in a fluorescent plate reader (excitation 320 nm, emission 405 nm) every 10 min for 70 minutes. Relative fluorescence units were normalized to protein concentration of the lysates [20].

### In Vitro Growth Assays

1×10^3^ cells were grown in flat bottom 96 well plates in 0.2 ml tissue culture medium. After 2–4 days of growth in some experiments viable leukemia cell population numbers were measured by an MTT assay (Molecular Probes) according to the manufacturer’s instructions. In other experiments viable cell numbers were measured by quantitative flow cytometry. All medium in a well was transferred to a flow sample tube and fluorescent microbeads (4×10^3^ per well, Flow Cytometry Absolute Count Standard, Bangs Laboratories) were added. Viable cells were identified by scatter characteristics and GFP-positivity (FL1), and cell numbers calculated as: (Total viable leukemia cells) = (Viable leukemia cells counted)×(4000/fluorescent microbeads counted). Wells were plated in triplicate.

### Matrigel Invasion Assay

Matrigel (BD Biosciences) (1 mg/ml, 100 µl) was added to the upper chamber of 24 well transwell tissue culture plate (Corning). 1×10^5^ leukemia cells in 100 µl RPMI with 1% fetal calf serum (FCS) were added to the upper chamber. The lower chamber contained 600 µl RPMI with 20% FCS. Following 24 hr incubation at 37°C cells in the lower chamber were counted. In some assays doxycycline was added to the medium at concentrations of 5 µg/ml or 10 µg/ml.

### Statistics

Student’s two-tailed t tests were used to compare means for normally distributed data. Wilcoxon tests were used to compared medians of nonnormally distributed data. A conventional two-tail p<0.05 was used to define statistical significance. Comparative survivals were analyzed using Kaplan-Meier graphs and logrank tests. Statistical calculations were performed using GraphPad Prism 4, Microsoft Excel 2007, and R: A language and environment for statistical computing. R Foundation for Statistical Computing, Vienna, Austria, http://www.R-project.org/.

## Results

### A Clone of the Acute Lymphoid Leukemia NSTY with Stable High Level Expression of Ly6a/Sca-1 is More Aggressive in Vivo than a Stable Low Ly6a/Sca-1 Clone

**Figure 1:**
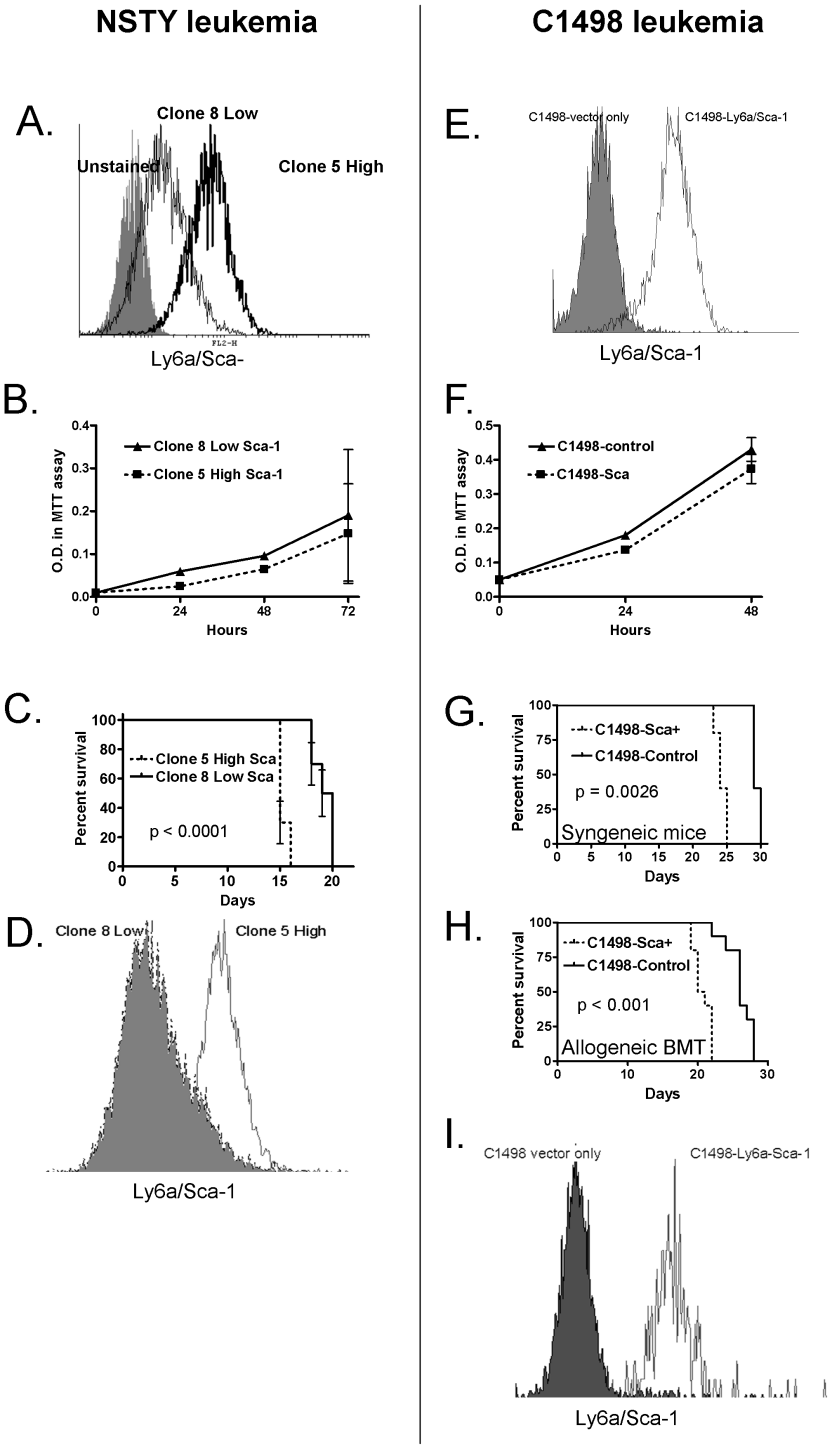
Leukemia cells expressing higher levels of Ly6a/Sca-1 grow more aggressively in vivo and lead to earlier death. Panels A – D depict experiments with NSTY, while panels E – I represent C1498. NSTY. (A) Flow cytometry for Ly6a/Sca-1 expression in clones 5 and 8 at the time of challenge. (B) In vitro growth of NSTY leukemia clones measured in MTT assays. Wells were plated in triplicate. Error bars represent standard deviation. P0.72 by t test. (C) NSTY-clone 5 which expresses high levels of Ly6a/Sca-1 grows more aggressively in vivo in syngeneic mice. Survival curves are presented. N = 10 per group. Animals challenged with NSTY clone 5 with high levels of L6a/Sca-1 expression had significantly shorter survival (p<0.001 by logrank test). (D) Ly6a/Sca-1 expression on NSTY clonal leukemias reisolated from mice in vivo. At time of euthanasia marrow samples were collected from mice and analyzed by flow cytometry for Ly6a/Sca-1 expression. Cells were gated on the leukemia blast population as defined by forward scatter and GFP expression. The filled histogram is the NSTY clone 8 low Ly6a/Sca-1 leukemia while the unfilled histogram is the clone 5 high Ly6a/Sca-1 leukemia. Representative examples are presented. C1498. (E) Flow cytometry compares surface Ly6a/Sca-1 expression on C1498-Ly6a/Sca-1 to that on control C1498-vector only cells. (F) In vitro growth of C1498-Ly6a/Sca-1 and C1498-vector only control cells as assessed by MTT assays. Wells were plated in triplicate. Error bars represent standard deviation. P0.15 by t test. (G) C1498-Ly6a/Sca-1 grows more aggressively in syngeneic mice. Syngeneic C57BL/6 mice were intravenously injected with 1×10^5^ leukemia cells and survival monitored. N = 5 per group. Mice challenged with C1498-Ly6a/Sca-1 exhibited significantly shorter survival (p0.0026 by logrank test). (H) C1498-Ly6a/Sca-1 grows more aggressively in allogeneic bone marrow transplant recipients. C57BL/6 mice underwent allogeneic bone marrow transplant using C3.SW donors. 1×10^6^ C1498 leukemia cells were mixed with the allogeneic hematopoietic cells and both were infused iv together. N = 10 per group. Mice receiving C1498-Ly6a/Sca-1 leukemia exhibited significantly shorter survival (p<0.001 by logrank test). (I) Ly6a/Sca-1 expression on C1498 leukemias reisolated from mice in vivo. At time of euthanasia marrow samples were collected from mice and analyzed by flow cytometry for Ly6a/Sca-1 expression. Cells were gated on the leukemia blast population as defined by forward scatter and GFP expression. The filled histogram is the C1498-vector only leukemia while the unfilled histogram is the C1498-Ly6a/Sca-1 leukemia. Representative examples are presented.

**Figure 2:**
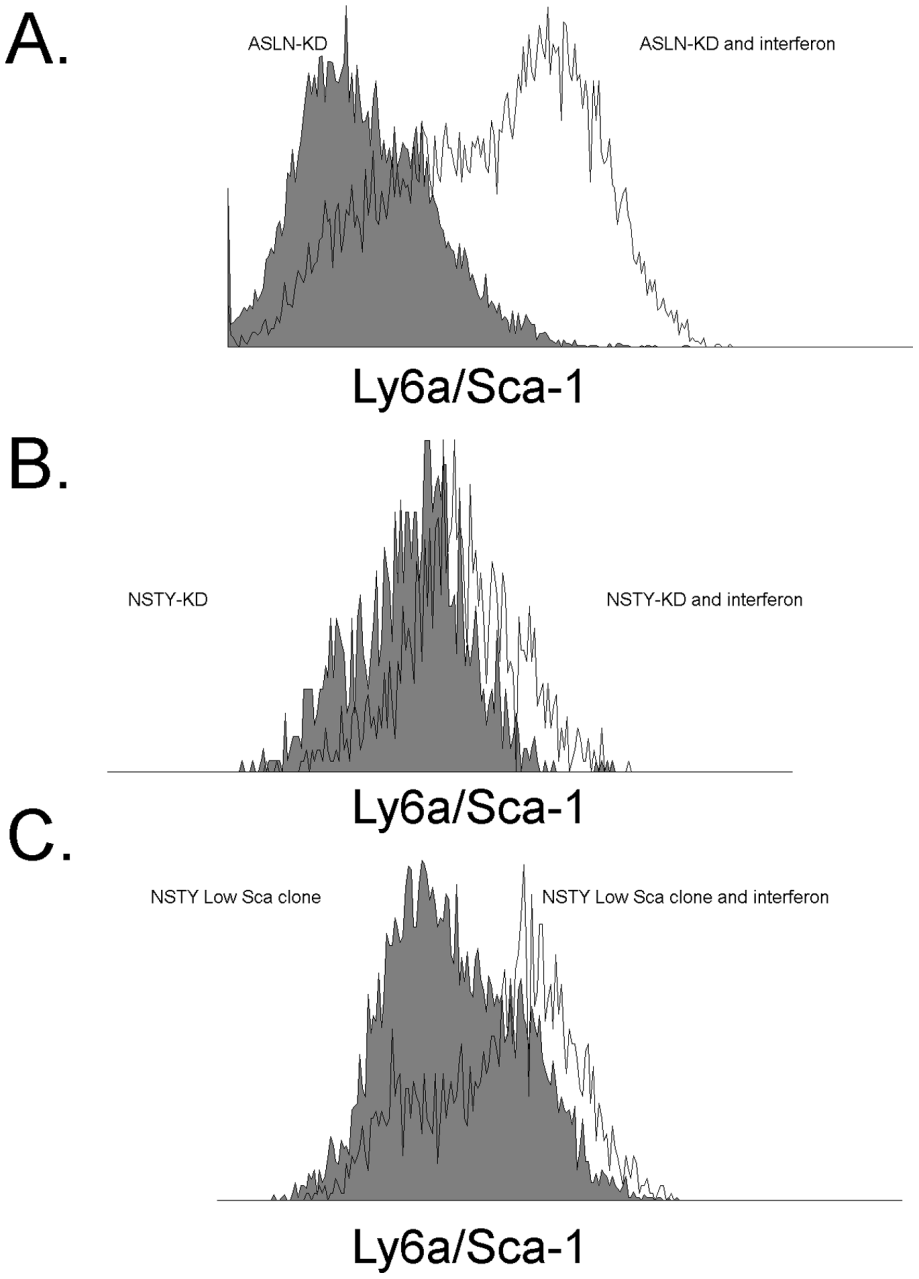
Higher levels of Ly6a/Sca-1 expression can be induced by interferon-gamma in Ly6a/Sca-1-knockdown leukemias and in low Ly6a/Sca-1 clones. Cells were treated with interferon-gamma 10 ng/ml for 24 hours. Controls were cultured 24 hours without interferon-gamma. Ly6a/Sca-1 was measured by flow cytometry. Filled histograms are controls without interferon while the unshaded histograms are the leukemias treated with interferon-gamma. (A) ASLN-lentiviral-Ly6a/Sca-1 knockdown. (B) NSTY-lentiviral-Ly6a/Sca-1 knockdown. (C) Low Ly6a/Sca-1 NSTY clone 8.

The acute lymphoblastic leukemia line NSTY expresses Ly6a/Sca-1. We performed limiting dilution cloning to isolate naturally occurring NSTY variants with different stable Ly6a/Sca-1 expression. Two stable clonal lines with distinctive Ly6a/Sca-1 expression were generated (Figure 1A). In vitro growth was not different (Figure 1B). Growth in vivo was examined and we observed that 0animals that were challenged with the leukemia that had higher levels of Ly6a/Sca-1 expression exhibited shorter survival compared to those challenged with leukemia cells with low Ly6a/Sca-1 expression (Figure 1C). At time of euthanasia leukemia in marrow was analyzed and demonstrated that levels of Ly6a/Sca-1 were stable in vivo in this experiment (Figure 1D). We chose not to repeat the cloning procedures to generate additional clones with widely differing Ly6A/Sca-1 because we observed that in nearly all cases Ly6a/Sca-1 levels remained inducible (Figure 2C).

### shRNA Mediated Knockdown of Ly6A/Sca-1 Expression in Leukemias that Express Ly6A/Sca-1 Decreases in Vivo Aggressiveness

**Figure 3:**
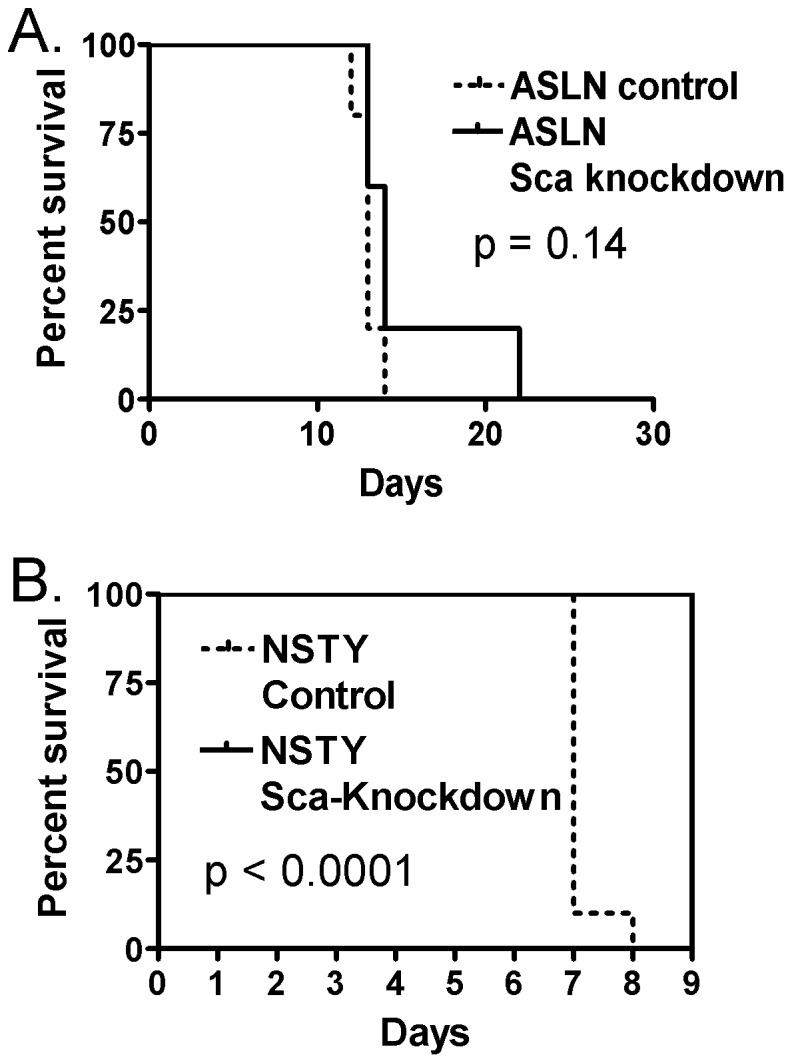
Comparison of in/Sca-1 knockdown leukemias. Mice were injected iv with 1×10^6^ leukemia cells and monitored daily for survival. “Control” were leukemia lines transduced with a control lentiviral vector containing only a puromycin resistance gene, while the “Sca-knockdown” had been transduced with a lentivirus containing an anti-Ly6a/Sca-1 sequence as well as the puromycin resistance gene. (A) ASLN leukemia. N5 per group. Control group had median survival of 13 days, while Sca-1 knockdown had median survival of 14 days. P0.14 by logrank test. (B) NSTY leukemia. N10 per group. Control group had median survival of 7 days while the Sca-knockdown had median survival of 9 days. P<0.0001 by logrank test.

To further test the relationship we used anti-Ly6A/Sca-1 shRNA lentiviral vectors to knockdown Ly6A/Sca-1 expression in two independent lymphoid leukemias that express high levels of Ly6A/Sca-1. ASLN and NSTY are B lineage murine acute lymphoid leukemias that express Ly6A/Sca-1. Knockdown was moderately effective with specific shRNA treated cells exhibiting approximately 60–70% less Ly6A/Sca-1 as assessed by flow cytometry (data not shown). Effective knockdown of the gene was transient. In troubleshooting this we observed that Ly6a/Sca-1 remained inducible even in those cells expressing the knockdown vector. Figures 2A and 2B demonstrate substantial increases in Ly6a/Sca-1 in both the NSTY and ASLN shRNA knockdowns when exposed to interferon-gamma, a cytokine commonly present in vivo. This observation suggested that in vivo Ly6a/Sca-1 expression in the knockdowns might be induced and make it difficult to see a difference in survival between the Ly6a/Sca-1 knockdown and controls. Nonetheless we challenged mice with knockdowns and controls from both the NSTY and ASLN leukemias. Figure 3A demonstrates for ASLN median survival in the control group was 13 days compared to 14 days in the Ly6a/Sca-1 knockdown. There were 5 animals in each group and the p value was 0.14. Figure 3B demonstrates that for the NSTY leukemia median survival in the control group was 7 days compared to 9 days in the Ly6a/Sca-1 knockdown. There were 10 animals in each group and the p<0.0001.

### Retroviral Transfer of the Ly6a/Sca-1 Gene to a Leukemia Line that Does not Express Ly6a/Sca-1 Leads to More Aggressive Growth in Vivo

While the results of experiments with naturally occurring variants and knockdowns were consistent with Ly6a/Sca-1 expression being associated with more aggressive growth in vivo we sought another experimental leukemia system in which differences in Ly6a/Sca-1were more distinct and stable. C1498 is an NKT cell leukemia of C57BL/6 origin that does not express Ly6a/Sca-1 on its cell surface either in normal conditions or when stimulated with interferon-gamma. C1498 cells were treated with a retroviral vector that encodes Ly6a/Sca-1 and also contains zeocin-resistance and green fluorescent protein (GFP) genes. Following zeocin selection a stable polyclonal line was produced that expressed high levels of Ly6a/Sca-1 (Figure 1E). As a control leukemia line C1498 was also treated with a control vector that contained only the zeocin-resistance and GFP genes. Expression of the Ly6a/Sca-1 gene did not change the in vitro growth rate of the leukemia (Figure 1F). However, in vivo growth was affected. Normal C57BL/6 mice were challenged with either C1498-Ly6a/Sca-1 or control C1498. Significantly shorter survival was observed in animals challenged with the Ly6a/Sca-1 expressing leukemia (Figure 1G). Flow cytometric analysis at time of euthanasia confirmed that Ly6a/Sca-1 expression was stable in vivo (Figure 1I). The experiment was repeated but with animals that were undergoing allogeneic bone marrow transplantation. The rationale for this was that our original observation of increased Ly6a/Sca-1 expression was in animals undergoing allogeneic transplant and we wished to confirm the effect in this setting. Again significantly shorter survival was seen in animals challenged with the Ly6a/Sca-1 expressing leukemia (Figure 1H).

**Figure 4:**
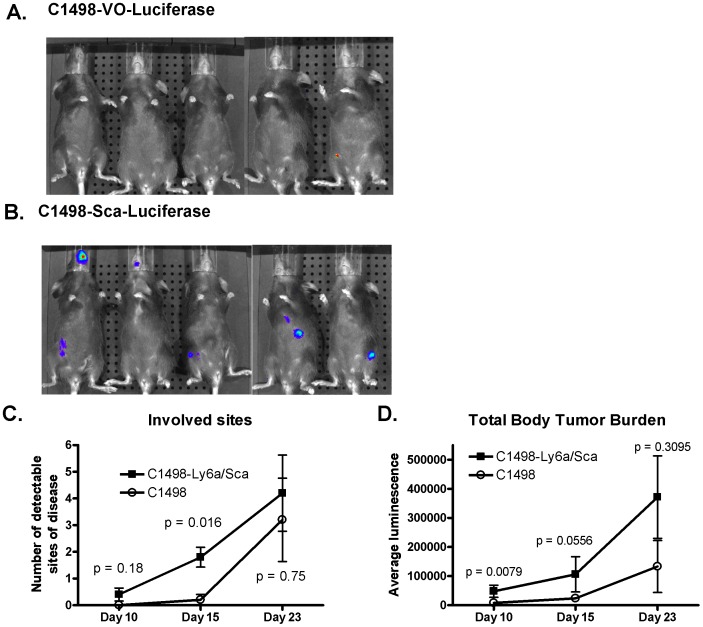
In vivo bioluminescent imaging demonstrates that C1498-Ly6a/Sca-1 disseminates more rapidly and creates greater total leukemia burden than the C1498-vector only control. Normal mice were intravenously injected with leukemia cells (5×10^5^) that had been transduced with a luciferase gene. Animals were imaged on days 10, 15 and 23 after injection. 5 animals in each group. Bioluminescent images at day 15 of (A) C1498-vector only, and (B) C1498-Ly6A/Sca challenged mice. (C) Number of discrete anatomic sites exhibiting luminescence. Animals were imaged and two independent investigators identified the number of discrete sites of luminescence in each animal. Symbols are the average number of sites and bars are standard errors of the mean. P values were calculated with a Wilcoxon test. (D) Total body leukemia burden measured by in vivo bioluminescence. Animals were imaged on days 10, 15 and 23 after intravenous injection with leukemia cells. Total luminescence for entire body was measured in photons/sec. Symbols represent the mean and bars the standard error of the mean. P values were calculated with a Wilcoxon test.

To gain greater insight into the in vivo behavior of the leukemia cells we transferred a luciferase gene to both the C1498-Ly6a/Sca-1 and C1498 control cell lines. This allowed in vivo bioluminescent imaging of leukemia growth, facilitating both identification of the number of sites of disease as well as total body burden of leukemia (Figure 4A & 4B). C1498-Ly6a/Sca-1 positive cells appeared in greater numbers of sites of disease (Figure 4C) as well as total leukemia burden (Figure 4D), both observations being consistent with greater capacity of the Ly6a/Sca-1 leukemia to disseminate.

### Ly6a/Sca-1 Associated in Vivo Aggressiveness is Correlated with Higher Levels of Matrix Metalloproteinases

The biological functions of Ly6a/Sca-1 are not well understood and consequently there was no obvious explanation for the increased in vivo aggressiveness of leukemias with higher levels of Ly6a/Sca-1 expression. It has recently been reported that myoblasts from Ly6a/Sca-1 deficient mice exhibit defects in muscle regeneration and reduced activity of matrix metalloproteinases [20]. MMPs are known to have an important function in modifying extracellular matrix proteins, a function that conceivably could have an impact on invasiveness of leukemia cells. This suggested the hypothesis that Ly6a/Sca-1 high leukemia cells might have higher levels of MMPs which could enhance the capacity of the leukemia to invade tissues in vivo.

### Ly6a/Sca-1 Expression is Associated with Increased Expression of MMP9

**Figure 5:**
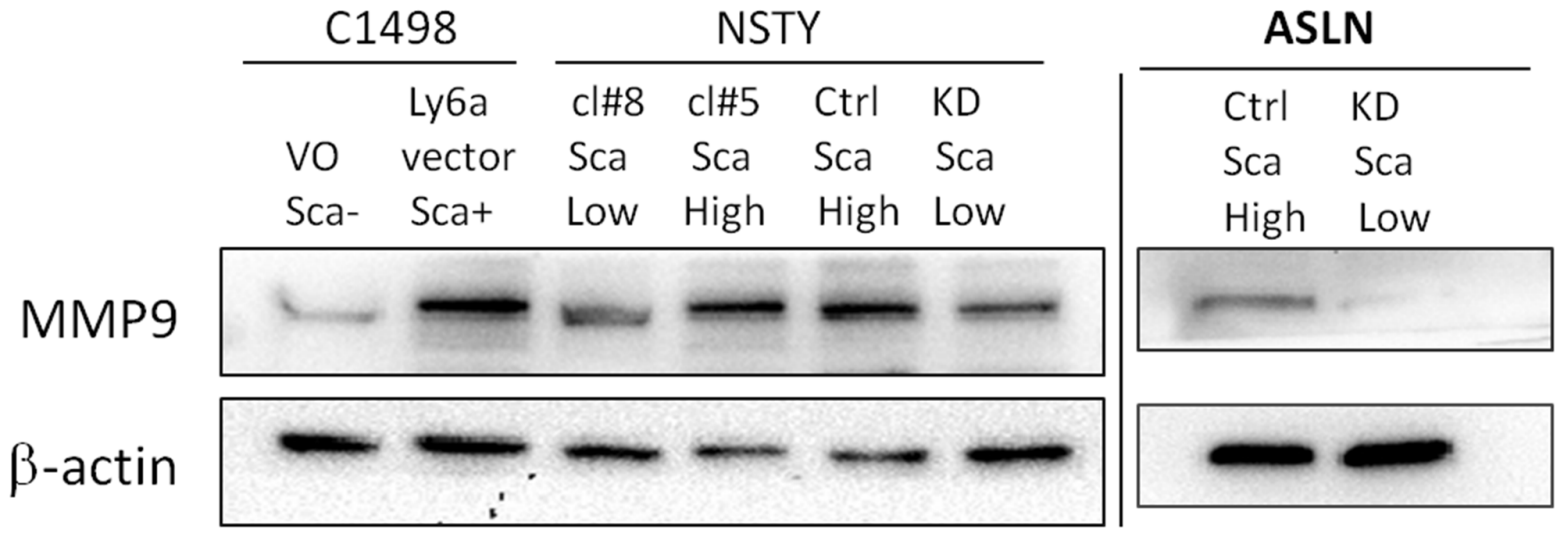
Higher levels of MMP-9 in Ly6a/Sca-1 expressing leukemias. Western blot for MMP-9. For the C1498 leukemia “VO Sca-” is the C1498-vector only control, while “Ly6a vector Sca+” is C1498-Ly6a/Sca-1. For the NSTY leukemia “cl#8 Sca Low” is clone 8 that has low levels of Ly6a/Sca-1, while “cl#5 Sca High” is clone 5 which has high levels of Ly6a/Sca-1. “Ctrl Sca High” is NSTY leukemia that was treated with a control shRNA vector, while “KD Sca Low” is NSTY after knockdown with an anti-Ly6A/Sca-1shRNA lentivirus vector. For ASLN “Ctrl Sca High” is ASLN leukemia that was treated with a control shRNA vector, while “KD Sca Low” is ASLN after knockdown with an anti-Ly6A/Sca-1shRNA lentivirus vector. ß-actin was used a control for assessing lane loading.

We explored this possibility with Western blot studies for MMP-9, a matrix metalloproteinase associated with metastatic capacity in malignancies. Figure 5 shows higher levels of MMP-9 protein in Ly6a/Sca-1 high leukemia cells. Lane 2 shows a much stronger band in C1498-Ly6a/Sca-1 line compared to the control C1498-vector only control. Lane 4 shows a stronger MMP9 signal in NSTY clone 5 that has higher levels of Ly6a/Sca-1 compared to clone 8 which has lower levels of Ly6a/Sca-1. Similar results were seen in both the ASLN and NSTY Ly6a/Sca-1 knockdown systems. Lane 6 shows a weaker MMP9 signal in the NSTY knockdown compared to its control in lane 5, while lane 8 shows a weaker MMP9 band in the ASLN knockdown compared to its control in lane 7.

### Ly6a/Sca-1 Expression is Associated with Increased Proteinase Activity

**Figure 6:**
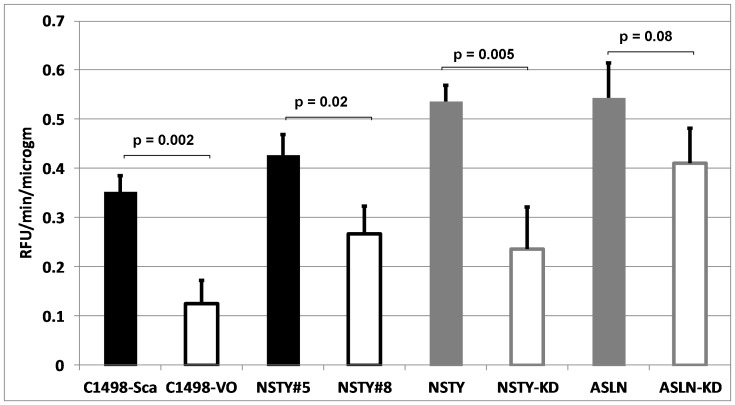
Greater metalloproteinase activity is observed in leukemia cells with greater expression of Ly6a/Sca-1. In vitro proteinase assays were performed in triplicate on cell lysates and expressed as relative fluorescence units per minute per microgram of protein. Filled bars represent leukemia lines with greater Ly6a/Sca-1 expression while white bars are those with low Ly6a/Sca-1 expression. “C1498-VO” is the C1498-vector only control, while “C1498-Sca” is C1498-Ly6a/Sca-1. “NSTY#8” is the NSTY leukemia clone 8 isolated by limiting dilution that has low levels of Ly6a/Sca-1 while “NSTY#5” is clone 5 which has high levels of Ly6a/Sca-1. “NSTY” is the NSTY line transduced with the lentivirus control vector while “NSTY-KD” is NSTY transduced with the anti-Ly6a/Sca-1 shRNA vector. For the ASLN leukemia “ASLN” and “ASLN-KD” have similar meanings. Bars are average and the error bars are standard variation. N = 3 per sample. P values for each comparison were calculated with t-tests.

We then extended this observation with a functional assay examining the capacity of leukemia cells to degrade a fluorogenic peptide which is known to be a substrate for a number of matrix metalloproteinases. Figure 6 (black bars and controls) demonstrates that Ly6a/Sca-1 high leukemia cells had significantly higher proteinase activity. Again, this relationship was consistent in all four experimental systems.

### Ly6a/Sca-1 Expression is Associated with Increased Invasiveness in Vitro

**Figure 7:**
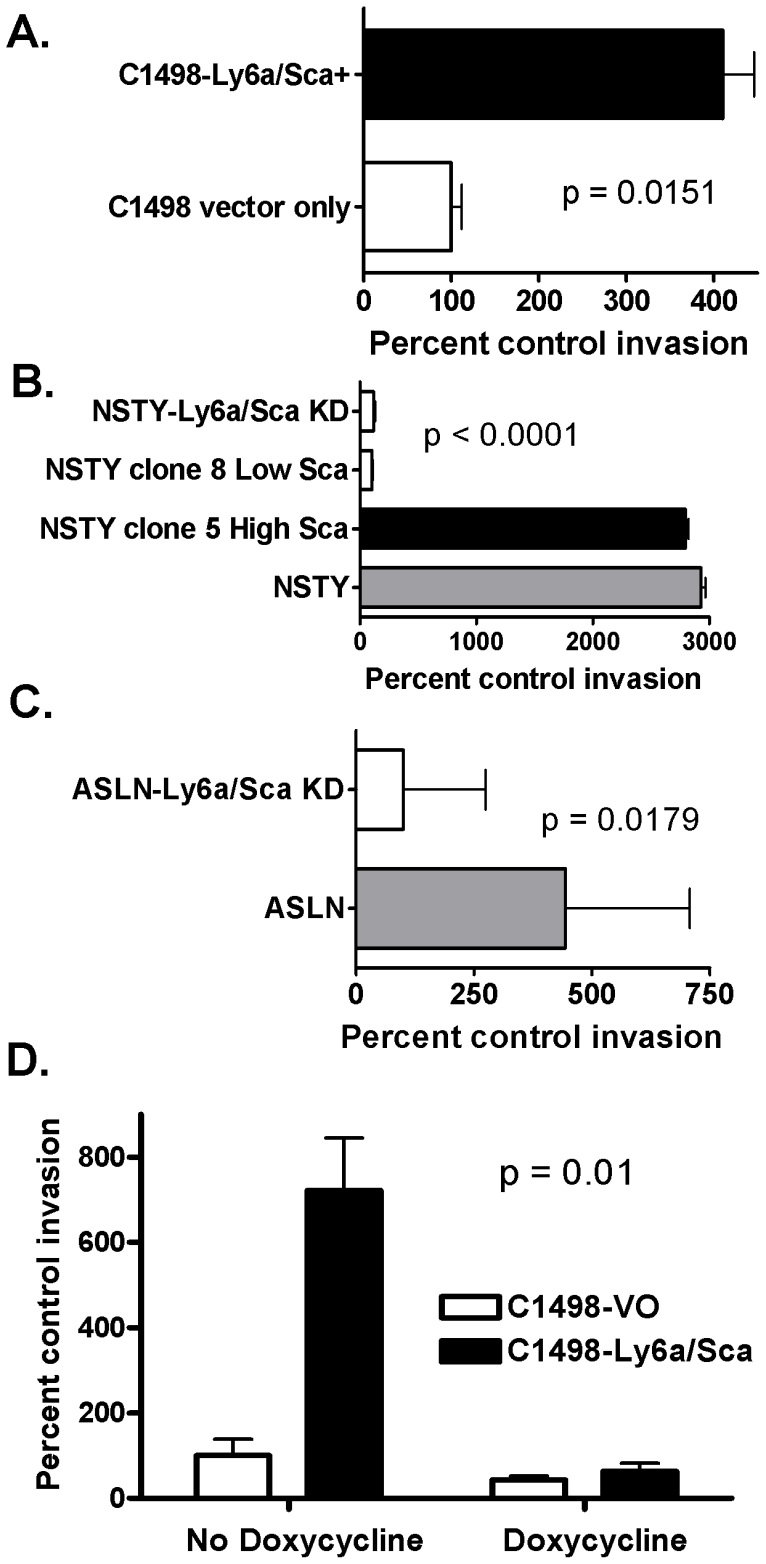
Leukemia cells with higher levels of Ly6a/Sca-1 expression exhibit greater invasiveness in Matrigel assays. In each assay the invasion of leukemia cells in the experimental group with higher levels of Ly6a/Sca-1 is expressed as percent relative to number of invading cells in the control group with low levels of Ly6a/Sca-1 expression. The control is represented as 100%. Averages and sem are displayed. (A) “C1498-Ly6a/Sca+” is C1498-Ly6a/Sca-1 compared to C1498-vector only. P0.0151 by t-test. N3 per group. (B) “NSTY” is the NSTY leukemia carrying the lentivirus control vector. “NSTY-Ly6a/Sca KD” is NSTY transduced with the anti-Ly6a/Sca-1 shRNA vector. “NSTY clone 8 Low Ly6a/Sca” is the NSTY leukemia clone 8 isolated by limiting dilution that has low levels of Ly6a/Sca-1 while “NSTY clone 5 High Ly6a/Sca” “ is clone 5 which has high levels of Ly6a/Sca-1. One way ANOVA was performed and generated a p<0.0001 for comparison of all groups. Planned t-tests between clone 8 and clone 5 yielded p<0.001 and between NSTY and the knockdown yielded p<0.001. N = 3 per group. (C) “ASLN” is the ASLN line carrying the vector only, while “ASLN-Ly6a/Sca KD” is ASLN transduced with the anti-Ly6a/Sca-1 shRNA vector. P0.0179 by t-test. N = 3. (D) Effect of the metalloproteinase inhibitor, doxycycline, on invasiveness in vitro. Matrigel invasion assays using C1498-Ly6a/Sca+ and control C1498-vector only cells were performed in the presence or absence of doxycycline 10 µg/ml in the medium. N4 per group. Doxycycline significantly reduced invasiveness in the C1498-Ly6a/Sca leukemia (p0.0018 by t-test). Doxycycline did not significantly reduce invasiveness in the control C1498-vector only leukemia (p0.199 by t-test, n = 4).

We then tested the functional significance of the enhanced protease activity by measuring the invasiveness of the leukemia cells. Leukemia cells were placed in the upper chamber of a split well plate in which Matrigel served as a barrier to cell migration to the lower chamber. One day later leukemia cells in the lower chambers were counted. Figure 7 demonstrates that Ly6a/Sca-1 high leukemia cells exhibited significantly greater capacity to invade and migrate through the Matrigel matrix. We observed this in all four leukemia models: the C1498 leukemia (Figure 7A), the NSTY leukemia clones (Figure 7B), the NSTY knockdown (Figure 7B) and the ASLN knockdown (Figure 7C).

### Increased Invasiveness was Abolished in the Presence of a Metalloproteinase Inhibitor

Doxycycline is an antibiotic that has been shown to exhibit inhibition of matrix metalloproteinases [21]. We hypothesized that if the increased in vitro invasiveness exhibited by the Ly6a/Sca-1 expressing leukemias were due to MMP activity, addition of doxycycline to the Matrigel invasion assays should abolish the increased invasion. Figure 7D demonstrates that this was indeed the case. Doxycycline can also be administered in vivo through drinking water and can affect metalloproteinase activity to some extent in vivo. We compared the growth of C1498-Ly6a/Sca-1 in animals administered doxycyline to those without the inhibitor. We observed a modest but statistically significant increase in median survival in doxycycline treated mice with C1498-Ly6a/Sca-1 (26 days versus 24 days, p0.0495 by logrank test, n = 5 per group). In vivo administration of doxycycline did not affect in vivo growth of control C1498 (median survival 28 days in both groups, p = 0.134, n = 5 per group).

## Discussion

Earlier work in our experimental system suggested the hypothesis that increased expression of Ly6a/Sca-1 would confer an advantage in vivo for leukemia cells [1]. Although the exact functions of Ly6a/Sca-1 remain unknown, the question was important since the gene is associated with the stem cell phenotype in many tissues including hematopoietic stem cells. To test this hypothesis we examined lymphoid leukemias that expressed either low or high levels of Ly6a/Sca-1. Mice were challenged with these high or low Ly6a/Sca-1 variants and survival was measured. Consistently we observed that leukemias that had higher levels of Ly6a/Sca-1 led to earlier death in these acute leukemia systems. We discovered that high Ly6a/Sca-1 leukemias had higher levels of matrix metalloproteinase activity. Our data suggest the hypothesis that earlier mortality with Ly6a/Sca-1 high leukemias could be due to the greater capacity of these cells to degrade and invade the extracellular matrix, ultimately producing more rapid dissemination of disease. We further speculate that this may be related to a potential normal role of Ly6a/Sca-1 in nonmalignant cells. Ly6a/Sca-1 was originally identified as an inducible activation molecule in lymphocytes and it is a credible hypothesis that Ly6a/Sca-1 may play facilitate activated lymphocytes entering and maneuvering within the extracellular matrix of tissues harboring infection.

Ly6a/Sca-1 is associated with a stem cell phenotype in a number of normal tissues (hematopoietic, hepatic, breast, muscle, among others) [14]. With the rise of the cancer stem cell hypothesis some have explored Ly6a/Sca-1 expression on cells capable of initiating cancers and have found Ly6a/Sca-1 present in the cancer initiating cells including chronic myelogenous leukemia [22], mammary carcinoma [23] and osteosarcoma [24]. In other studies Ly6a/Sca-1 expression in tumors has been correlated with a more aggressive phenotype, e.g., in mammary tumors [25], [26], retinoblastoma [27] and prostate cancer [28].

While these data do not prove that the enhanced matrix metalloproteinase activity is the mechanism of increased aggressiveness seen in Ly6a/Sca-1 high leukemia cells, they are consistent with a substantial body of research that does show MMP-9 activity is important in the determining the invasiveness of human chronic lymphocytic leukemia [29], [30]. Recent research also suggests that the effect of MMPs may go beyond degrading the extracellular matrix. Degradation of local chemokines and growth factors could be involved, as well as initiation of signaling pathways by MMPs interacting via their noncatalytic domains with receptors on leukemia cells [31]. Research in acute myeloid leukemia has suggested that increased MMP activity is associated with more aggressive human leukemias [32]. In solid tumor malignancies MMP activity has often been associated with greater invasiveness and metastatic capacity [33]–[36].

There are several limitations to this study. First, while we have identified a strong correlation between Ly6a/Sca-1 expression, metalloproteinase activity and increased in vivo aggressiveness, the mechanistic causal link has not been strictly proven. We have not specifically identified which metalloproteinases mediate the effect. Second, we cannot exclude altered cell cycle or cell growth kinetics as making some contribution to the in vivo phenotype in some of the models. Third, we cannot exclude the possibility that in our experimental systems the leukemias with higher levels of Ly6a/Sca-1 may include more “leukemia stem cells”. There are no well-defined flow cytometric phenotypes of leukemia stem cells in our experimental leukemias that would allow this to be ascertained in vitro. In vivo estimates of stem cell numbers by limiting dilution engraftment experiments would be confounded by the increased metalloproteinase activity which might increase the probability of engraftment of Ly6a/Sca-1 high expressing leukemia cells, producing an inaccurate estimation of leukemia stem cells. Fourth, since Ly6a/Sca-1 is a cell surface protein GPI-anchored protein in a complex lipid raft that interacts with several signaling pathways [14], [37] (e.g., Src family kinases, Lyn, c-Kit, Fyn) it is very likely that there are additional complex effects that contribute to the overall phenotype of increased in vivo aggressiveness. Fifth, while in the C1498 leukemia model Ly6a/Sca-1 expression levels were stable, in both the ASLN and NSTY leukemias levels of Ly6a/Sca-1 were not fixed over time, but appeared to vary, making experimental comparisons more challenging in these models. The mechanism of the variability is not fully known, but we did establish that interferon-gamma does induce the gene, compatible with the observation that Ly6a was first described as a lymphocyte activation marker. Sixth, while Ly6a/Sca-1 is a biologically important molecule in murine models for study of stem cell biology, it is important to note that there is no exact human homolog for it. However, Ly6a/Sca-1 is one member of a large Ly6 gene family most of which are GPI-anchored proteins, and there is considerable similarity between humans and mice in this family [38]. These human Ly6 family genes have not been well characterized although there is evidence that they are expressed in human hematopoietic cells [38]. In addition, several members of the Ly6 family have been shown to be important in a variety of human cancers [39]–[41].

In summary, this work examined the potential role of Ly6a/Sca-1, a molecule that is closely associated with the stem cell phenotype in many murine tissues, in contributing to the aggressiveness of lymphoid leukemias in vivo. We found that increased expression of Ly6a/Sca-1 lead to more rapid progression of lymphoid leukemias in vivo. In addition, we found that there was a relationship between Ly6a/Sca-1 expression and expression of matrix metalloproteinases, providing one plausible explanation for the increased aggressiveness. Our ongoing work will explore whether the related human Ly6 family of genes produce similar changes in leukemia behavior.
